# Editorial: Naturally occurring compounds and their applications in endocrinology: mechanisms, therapeutic potential and clinical applications

**DOI:** 10.3389/fendo.2026.1821832

**Published:** 2026-03-19

**Authors:** Ramith Ramu, Shashank M. Patil

**Affiliations:** Department of Biotechnology and Bioinformatics, School of Life Sciences, JSS Academy of Higher Education and Research (JSS AHER), Mysuru, Karnataka, India

**Keywords:** immunometabolism, insulin resistance, natural bioactive compounds, polycystic ovary syndrome (PCOS), programmed cell death

Endocrine and metabolic conditions, including polycystic ovary syndrome (PCOS), obesity-associated insulin resistance, diabetes mellitus, acute pancreatitis, and neurobehavioral comorbidities such as chronic pain and depression, are an increasing public health burden. These disorders frequently share overlapping biology, including persistent low-grade inflammation, redox imbalance, altered energy metabolism, and disrupted intracellular signaling (1). They also cluster clinically because key mechanistic hubs, such as impaired insulin signaling, mitochondrial stress, and immune activation, connect endocrine and metabolic dysfunction across organ systems (2). In acute inflammatory settings, dysregulated programmed cell death can further intensify tissue damage and systemic responses. This Research Topic, “Naturally Occurring Compounds and Their Applications in Endocrinology: Mechanisms, Therapeutic Potential and Clinical Applications,” highlights how naturally occurring bioactives may act on shared pathways to support endocrine and metabolic homeostasis.

PCOS is a central theme across several contributions, consistent with its multifactorial pathophysiology and high prevalence. Balkrishna et al. synthesize evidence indicating that apigenin and ellagic acid may target multiple PCOS features, with reported anti-inflammatory, antioxidant, insulin-sensitizing, and anti-androgenic activities relevant to hyperandrogenism, insulin resistance, and ovarian dysfunction. The review links these effects to changes in ovarian steroidogenesis, modulation of PI3K/Akt signaling, inhibition of 5α-reductase, and reduced pro-inflammatory cytokine signaling. It also discusses formulation approaches, including nanoformulations and phytosomes, as practical routes to address bioavailability constraints.

In a complementary review, Chang et al. summarize how resveratrol may influence endocrine and metabolic axes in PCOS. They outline pathways through which resveratrol could support reproductive and metabolic balance, with emphasis on improvements in glucose and lipid handling and attenuation of oxidative stress. Together, these PCOS-focused articles reinforce that clinically relevant benefit is more likely when interventions address metabolic dysfunction alongside reproductive manifestations, with insulin resistance and inflammation as recurring therapeutic nodes.

Extending this framework, Sun et al. review PCOS complicated by insulin resistance and discuss the integrative role of Traditional Chinese Medicine (TCM). They summarize evidence that multi-component interventions may reshape gut microbiota, dampen inflammatory cytokine output, modulate androgen-related dysregulation, and improve insulin signaling. Importantly, the authors also emphasize practical barriers to translation, including variability in preparations, standardization challenges, and the need for well-designed clinical protocols to convert promising signals into evidence-based care.

Obesity and insulin resistance, major drivers of endocrine complications, are addressed from both mechanistic and systems perspectives. Das et al. compile data suggesting that polyphenols, alkaloids, and marine carotenoids can influence adipogenesis, thermogenesis, and metabolic flexibility through pathways that include PPARγ, C/EBPα, AMPK, and UCP1. They also discuss potential links to gut-derived metabolites and note that reported clinical signals, such as reduced adiposity and metabolic improvement, support continued evaluation of multi-target strategies for complex metabolic disease.

Barrera-Esparza et al. extend this theme with network-based modeling of adipose tissue regulation in insulin resistance. By integrating inflammatory and insulin-signaling modules, they identify candidate regulatory nodes and bioactives, including anthocyanins, punicalagin, oleanolic acid, and NRG4, that could facilitate transitions toward a metabolically healthier state. This approach underscores the value of prioritizing targets within interacting networks rather than relying on single-pathway inhibition.

Diabetes and its complications represent another major axis of the Research Topic. Zhang et al. review procyanidins and describe preclinical evidence for antioxidant, anti-inflammatory, and antihyperglycemic actions associated with improved insulin sensitivity and endothelial function. By framing vascular dysfunction and oxidative stress as shared determinants of disease progression, the review positions procyanidins as candidates relevant to complications such as nephropathy, retinopathy, neuropathy, and cardiovascular disease, aligning with the broader focus on immunometabolic drivers across the Research Topic.

The inclusion of acute pancreatitis broadens the scope from chronic metabolic dysregulation to acute pancreatic injury with endocrine implications. Liu et al. review programmed cell death (PCD) signaling in acute pancreatitis and highlight apoptosis, autophagy, pyroptosis, and ferroptosis as determinants of acinar cell fate and systemic inflammatory escalation. Their synthesis suggests that plant-derived natural products may modulate PCD pathways, offering mechanistically grounded entry points for therapeutic development.

Two original research studies further extend the mechanistic narrative into neuroendocrine and pain-related phenotypes. Huang et al. report that kaji-ichigoside F1 activates PPAR-γ/CX3CR1/Nrf2 signaling while suppressing NF-κB/NLRP3 pathways, with associated mitigation of LPS-induced depression-like behaviors, consistent with a recurring emphasis on inflammatory control and redox balance. Wang et al. examine YB60 derived from Corydalis Rhizoma and Paeoniae Radix Alba and demonstrate analgesic efficacy linked to inhibition of AR/Mboat2-mediated ferroptosis in spinal cord neurons. Together with the pancreatitis review, these studies highlight ferroptosis- and inflammasome-linked signaling as shared immuno-metabolic threads across inflammatory, degenerative, neuroendocrine, metabolic, and pain-related phenotypes.

Collectively, the contributions in this Research Topic support the view that endocrine and metabolic disorders arise from interacting networks that couple inflammation, oxidative stress, insulin signaling, mitochondrial function, lipid metabolism, and regulated cell death. Naturally occurring compounds discussed here frequently show multi-target activity across these hubs. However, persistent challenges remain, including bioavailability limitations, inconsistent standardization, dosing variability, and gaps in translational validation. Addressing these issues through improved formulation strategies and rigorously designed clinical studies will be critical for moving from mechanistic plausibility to clinically meaningful impact.

By combining mechanistic reviews, systems-level modeling, and preclinical experimental evidence, this Research Topic reflects the growing maturity of natural product research in endocrinology. The Research Topic highlights opportunities for precision and multi-target approaches that bridge ethnopharmacological knowledge with mechanistic depth and clinical standardization.
